# Exosomes: a new perspective in EGFR-mutated lung cancer

**DOI:** 10.1007/s10555-021-09962-6

**Published:** 2021-04-14

**Authors:** Amina Jouida, Cormac McCarthy, Aurelie Fabre, Michael P. Keane

**Affiliations:** 1 UCD School of Medicine, UCD Conway Institute of Biomolecular and Biomedical Research, University College Dublin, Dublin, Ireland; 2 St. Vincent’s University Hospital and School of Medicine, University College Dublin, Dublin, Ireland

**Keywords:** Exosome, EGFR, Biomarker, Lung cancer

## Abstract

Exosomes are major contributors in cell to cell communication due to their ability to transfer biological material such as protein, RNA, DNA, and miRNA. Additionally, they play a role in tumor initiation, promotion, and progression, and recently, they have emerged as a potential source of information on tumor detection and may be useful as diagnostic, prognostic, and predictive tools. This review focuses on exosomes from lung cancer with a focus on EGFR mutations. Here, we outline the role of exosomes and their functional effect in carcinogenesis, tumor progression, and metastasis. Finally, we discuss the possibility of exosomes as novel biomarkers in early detection, diagnosis, assessment of prognosis, and prediction of therapeutic response in EGFR-mutated lung cancer.

## Introduction

Lung cancer is one of the primary causes of death from cancer around the world, the prognosis of which is poor (5-year survival rate of 10%). It is possible to categorize lung cancer into two broad groups: small cell lung cancer (SCLC), which accounts for approximately 15% of the total number of cases, and non-small cell lung cancer (NSCLC), which accounts for around 85%. NSCLC is divided into three primary types: adenocarcinoma (representing around 50% of all NSCLC), squamous cell carcinoma (which accounts for approximately 30%), and large-cell undifferentiated carcinoma (comprising around 15%). Surgical resection is the preferred treatment option for a tumor early in its growth, although tumor recurrence and metastasis are commonly used, even after resection, with morphological features such as papillary or solid architecture correlating with poor survival and altered response to chemotherapy [1].

Comprehending the biological mechanisms underlying the development of tumors as well as possible biomarker expression of cancer is critical for precise diagnosis at an early stage, targeted therapy, and the development of medications. Large-scale collaborative works have facilitated the understanding of genomic modifications in tumors, hence enabling possible diagnostic and therapeutic targets to be identified, one of which is the epidermal growth factor receptor (EGFR). For patients with adenocarcinoma, EGFR mutations are now identified when the diagnosis is made. Such a mutation in lung adenocarcinoma has a prevalence that ranges from 10 to 78%, with significant differences according to ethnic origins and geographical locations [2]. The development of EGFR tyrosine kinase inhibitors (EGFR-TKIs) represents considerable progress for the treatment of lung cancer, and they are now recognized first-line treatments for NSCLC patients who have activating EGFR mutations (within exon 19 and the L858R missense mutation within exon 21) [3]. The identification of mutations within the *EGFR* gene in tumor tissues is acknowledged to be the most effective method of estimating the efficiency of treating NSCLC with EGFR-TKIs. Nevertheless, in up to 20% of patients, tissue biopsies are either not informative or not available due to the lack of sufficient neoplastic tissue or because it is technically infeasible [4]. Additionally, only a limited number of cancers are diagnosed at an early stage, which emphasizes the necessity for early detection and targeted treatment. Consequently, liquid biopsy has the potential to become a complementary/alternative tool for diagnosis and prognosis to conventional tissue biopsy. This method comprises the detection of proteomic and genomic information from body fluids, specifically samples of blood. Moreover, this approach has promise for clinical practice due to its noninvasiveness and easy repeatability. The assessment of a liquid biopsy provides a genetic overview of all cancerous lesions (primary and metastases) [5, 6]. Circulating free DNA (cfDNA), exosomes, and circulating tumor cells (CTCs) can potentially be used for noninvasive screening, diagnosis, and prognosis, as well as evaluating treatment response [7–9]. Additionally, liquid biopsy facilitates the real-time monitoring of disease and the biological responses of the patient undergoing treatment [10].

A growing body of evidence has shown that exosomes can provide important information regarding tumors because of their capability to transfer DNA, RNA, and proteins between cells. Furthermore, in lung cancer harboring EGFR mutations, findings indicate that exosomes represent suitable biomarkers for research into cancer and clinical application while also revealing new pathways in the discipline of nanomedicine. In this review, the function of exosomes in lung cancer is discussed, with a specific focus on lung cancers harboring EGFR mutations. We describe the contribution of exosomes to carcinogenesis, the progression of tumors, and metastasis development. Lastly, we review the potential of exosomes to be used as novel biomarkers for the purpose of diagnosing cancer at an early stage, in addition to assessing prognosis, and as therapeutic predictors in EGFR-mutated lung cancer.

## The role of exosomes

In 1987, Johnstone et al. [11] first defined the term “exosomes” for a class of extracellular vesicles (EVs), which Pan et al. [12] initially described in 1985. EVs are grouped into three main categories according to their magnitude and assumed biogenetic pathways; the first is apoptotic bodies that have a diameter ranging from 800 to 5000 nm and are emitted by cells experiencing programmed cell death. The second type is microvesicles, which are large membranous vesicles (with a diameter of 50–1000 nm) that are generated via budding from the membrane of the plasma. The last category is exosomes, which are vesicles with a diameter of 30–100 nm regarded as having endocytic origin and include a lipid bilayer membrane [13].

All types of cells can release exosomes, such as astrocytes, epithelial cells, neurons, hematopoietic stem cells, fibroblasts, adipocytes, melanocytes, mesothelioma cells, and tumor cells [14–16]. Exosomes have high heterogeneity and are likely reflective of the phenotypic condition of the cell from which they are generated. It is possible to detect exosomes in a variety of biological fluids including plasma, serum, semen, breast milk, urine, nasal secretions, saliva, amniotic fluid, cerebrospinal fluid, pleural, and ascitic fluid [17–19]. Exosomes contain proteins, lipids, and RNA (such as mRNA, microRNA [miRNA], and additional noncoding RNA) [20–24]; among those proteins are different human epidermal growth factor receptors [25–27]. Additionally, cytokines, transcription factor receptors, growth factor receptors, and different bioactive molecules are contained in exosomes. In 2019, Jeppesen et al. [28] demonstrated that exosomes do not carry double-stranded DNA (dsDNA) or DNA-binding histones. Exosomal proteins like the tetraspanins (CD63, CD81, CD9), TSG101, Alix, and Heat Shock Protein-70 (HSP-70) are regarded as exosomal markers and are employed for the purpose of identifying the existence of vesicles as verifiable exosomes [20, 28–30]. The lipid components of exosomes include cholesterol, sphingomyelin, hexosylceramides, phosphatidylserine, and saturated fatty acids, which all constitute the plasma membrane [31].

It is estimated that normal human blood contains approximately 2000 trillion exosomes, whereas the number of exosomes in the blood of cancer patients is around 4000 trillion [32–34]. Rabinowits et al. [35] conducted a study in which they investigated the extent of circulating tumor exosomes in the plasma of two groups composed of 27 lung adenocarcinoma patients and 9 healthy controls. The findings indicated there were more exosomes in adenocarcinoma patients (mean 2.85 mg/mL) in comparison to the controls (mean 0.77 mg/mL). It was theorized that the higher level of exosomes in diseased patients was caused by alterations to cellular physiology. Studies also revealed that circulating exosomes were more concentrated in patients with breast, ovarian, and pancreatic cancer [36, 37], which could increase their potential to be used as biomarkers for malignancies. Various researchers have concentrated on the underlying mechanism via which exosomes are upregulated in lung cancer. For instance, Yu et al. [38] described that exosome production by cells was found to be regulated by tumor protein p53. In a different study, it was shown that YKT6, which belongs to the soluble *N*-ethylmaleimide-sensitive-factor attachment receptor (SNARE) family of proteins, is a molecule that plays a critical role in the regulation of exosome release in lung cancer cells [39]. Exosome function depends on their parental cytotypes and contents; exosomes derived from normal cells contribute to the process of sustaining stable homeostasis, while exosomes derived from tumor cells are associated with the progression of the tumor. The identification of specific exosomal markers to discriminate stages and/or histological subtypes in lung cancer is still ongoing. Nonetheless, one study has identified specific miRNA profiles for adenocarcinoma and squamous cell carcinoma [40]. Also, exosomal Tim-3 and Galectin-9, actors of immune surveillance suppression, have been shown to correlate with squamous cell carcinoma and more malignant characteristics [41].

The exact physiological role played by exosomes is still unclear, although it is probable that they have some involvement in the removal of surplus and unneeded cell components, acting as a form of waste disposal; however, recent studies indicate that they also contribute to signaling between cells as a result of their capacity to carry RNA molecules and proteins from one cell to another. It has been recently shown that EVs with the ability to deliver cargos to target cells they meet could eventually cause such recipient cells to be reprogrammed distal from the EV release. Hence, exosomes provide a new means of communication between cells, which could be critical in numerous cellular procedures, including immune responses [42, 43], signal transduction [44], and antigen presentation [45]. Therefore, thanks to their biologically active cargos, exosomes could provide prognostic data on a variety of diseases, including chronic inflammation [46], cardiovascular and renal diseases [47, 48], neurodegenerative diseases [49–51], lipid metabolic diseases [52], and malignancy [53–55].

Exosome contents have a significant influence on how they affect recipient cells. It has been proposed that the miRNA contents of circulating exosomes have similarities to those in cancer cells from which they originated, highlighting the possibility of using miRNA in cancer diagnostics [56–58]. The ability to detect exosomal miRNA in bodily fluids demonstrates the possible benefits of exosomal miRNA as disease biomarkers [59]. Likewise, it is probable that exosome proteins mirror the properties of their origin cells and could indicate the potential for the development of metastasis [60, 61], as communication can occur between parental cells and particular distal or proximal target cells via exosome amplification [62]. Furthermore, the cell tropism of exosomes is specifically related to their cellular origin and properties, characteristics that can be utilized for targeting diseased tissues and/or organs, as suggested by Hoshino et al. [60], who showed that it is possible to use exosomal integrins for the prediction of metastasis in specific organs.

## Functional effect of EGFR-mutated exosomes

**Fig. 1:**
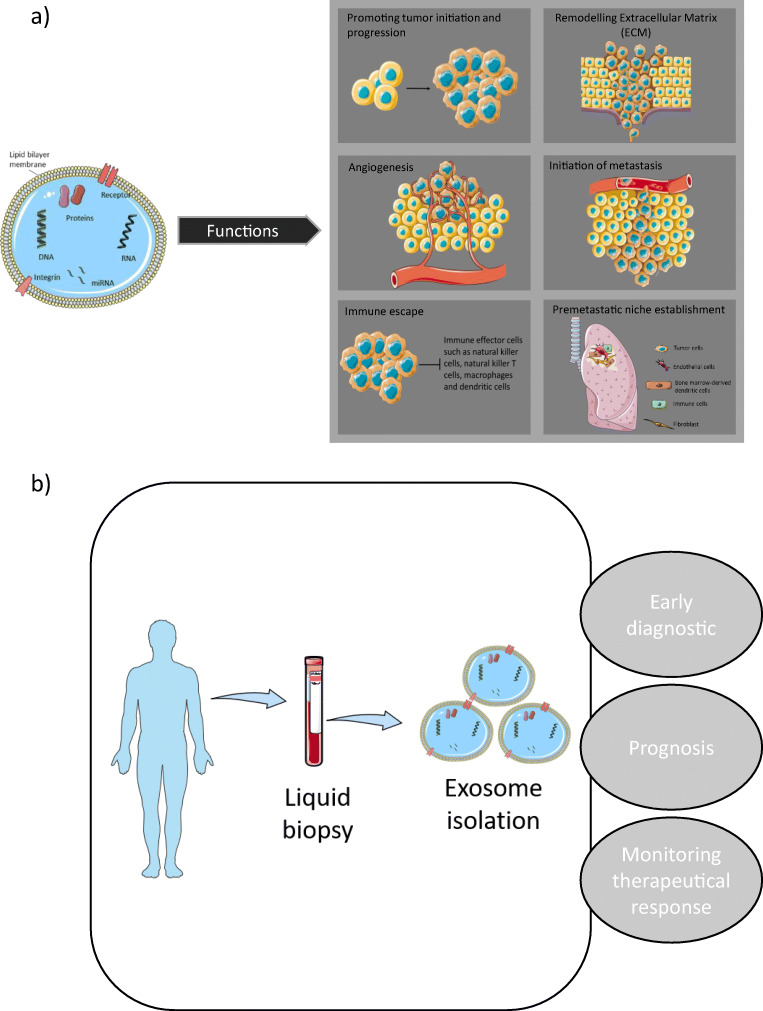
Exosomes display different functional roles **a** allowing potential use in the clinical application **b** in EGFR-mutated cancer

Receptor cells can take up exosomes emitted by donor cells in an autocrine, paracrine, or endocrine manner, thus demonstrating the pivotal role that exosomes play in intercellular communication [63, 64]. Transferring functional exosome content to receptor cells can lead to pathological or physiological effects. Within tumor cells, significant alterations occur to endosomal sorting complexes required for transport, which could cause considerable modifications to the exosomes’ molecular profile as well as the volume of exosomes emitted [29, 65]. An increasing amount of research is being conducted on exosomes as novel mediators of cell-to-cell communication for tumorigenesis as well as progression in the tumor microenvironment in many types of cancer, including lung cancer [66–68] (Fig. 1a). As it is possible to easily obtain and characterize exosomes from the majority of bodily fluids, they have significant potential to be applied in screening programs or as predictive/prognostic biomarkers and can also be used as possible liquid biopsy biomarkers for lung cancer [69]. Exosomes have the ability to transport molecules derived from tumors (DNA and RNA), and the findings of exosomal nucleic acid analysis reveal they have sensitivity in the identification of pertinent mutations [70, 71].

Zannetti-Domingues et al. [72] depict a major role of EGFR in exosome trafficking (biogenesis and uptake by recipient cells) thereby on its own emission (probably due to the involvement of exosomes in the activation of EGFR recycling mechanisms [73]) and signalization. It was observed that EGFR (as well as its oncogenic mutants) and its signaling network proteins were commonly expressed in lung cancer exosomes from varying sources [74–76]. EGFR mutation relevance in malignancies is well recognized [77]. In so doing, it is important to monitor and further understand the biology of exosomes carrying EGFR mutations.

### Promotion of tumor initiation and progression

It has been shown that microvesicles originating from glioma cells that express EGFR in its truncated form, EGFRvIII, deliver this oncogenic protein to neighboring cells in which it is not expressed [78]. This causes transforming signaling pathways to be activated as well as the regulation of target gene expression, such as vascular endothelial growth factor (VEGF), anti-apoptotic Bcl-x(L), and cyclin-dependent kinase inhibitor p27. Research has revealed that the transfer of EGFR could occur through exosomes from human carcinoma cell lines containing activated EGFR to endothelial cells, thus causing mitogen-activated protein kinase (MAPK) and AKT pathways to be activated as well as the expression of VEGF, which increases the capacity for anchorage-independent growth and anti-apoptotic gene expression [74]. Testing on EGFR localized to exosome membranes has been performed to evaluate its potential as a marker for diagnosing lung cancer [79]. For instance, EGFR derived from exosomes is a possible differentiating biomarker for diagnosing NSCLC and chronic lung inflammation. Huang et al. [75] determined that around 80% of exosomes from cancer biopsies were positive for EGFR, whereas a mere 2% of exosomes extracted from chronically inflamed lung tissue were EGFR positive. It was shown by Yamashita et al. and Huang et al. [75, 79] that exosomal EGFR protein can potentially be used as a biomarker to characterize lung cancer; nevertheless, the aforementioned studies had comparatively small sample sizes, and therefore, the findings should be corroborated by a larger cohort.

### Immune escape

The immune system interacts closely with tumor cells in the body. Immune escape is characterized by the ability of cancer cells to escape immune surveillance and antitumor immunity, which is a key mechanism of metastatic progression.

Other researchers have highlighted that exosomes derived from cancer mediate the interaction among tumor and immune cells to modify antitumor immunity. For instance, EGFR signaling within lung cancer cells can jeopardize antitumor immunity, which is caused by the transferral of EGFR to host macrophages by exosomes, which resultantly suppresses the innate antiviral immunity [80]. Yu et al. [81] investigated the role of the EGFR-containing exosomes on dendritic cells. The findings revealed that lung cancer with the EGFR E746-A750 deletion mutation caused the antitumor immunity to be repressed by dendritic cells via exosomes. It has additionally been demonstrated that stromal-derived exosomal-EGFR is a critical factor in balancing macrophage polarization and prostatic wound healing. Shi et al. [82] revealed that the production and secretion of exosomal-EGFR and other cytokines and chemokines, after shallow heat injury, by prostate stromal fibroblasts caused greater proliferation and migration of prostate epithelial cells as the wound healed. Exosomes derived from cancer cells that contain EGFR contribute to the progression of metastasis; Zhang et al. [83] showed that EGFR-containing exosomes could facilitate the creation of a liver-like microenvironment that promotes metastasis specific to the liver. They demonstrated that exosomes derived from gastric cancer transport EGFR to the liver and such EGFR are ultimately incorporated into liver stromal cells’ plasma membrane. Further *in vitro* experiments have shown that hepatocyte growth factor (HGF) is effectively activated by such translocated EGFR by the suppression of miR-26a/b, and the upregulated paracrine HGF, which binds the c-MET receptor onto migrated cancer cells, establishes a fertile microenvironment so that metastatic gastric cancer cells can land and proliferate.

### Angiogenesis

Angiogenesis is a critical factor in the process of a microscopic tumor expanding into a macroscopic tumor that has clinical relevance. Exosomes derived from cancer have been identified as novel contributors to angiogenesis [84]. The protein cargo and molecular properties of exosomes may be reflective of the hypoxic condition of the tumor and its aggressiveness [85]. Analogous findings were discovered for exosomes derived from lung cancer and leukemia cells. In addition to modified phenotype properties, exosomes derived from hypoxic lung cancer cells have been found to promote angiogenesis and increase endothelial cells’ permeability, thus triggering cancer cell intravasation/extravasation [86, 87]. VEGF is one of the pro-angiogenic proteins that is acknowledged to be an important initiator of angiogenesis [88]. It can be detected within serum exosomes of mice that have recurrent lung cancer in levels that are 213-fold greater than mice without lung tumors. It has been suggested that the poor prognosis and progression of tumors in 60% of NSCLC may be attributed to the overexpression of VEGF [89–91]. Another factor that motivates the exploration of EGFR as a possible target in cancer and angiogenesis is associated with the fact that, while this receptor is predominantly not present in standard endothelial cells, selective overexpression commonly occurs by the tumor-related vasculature [92, 93]. EGFR can be transferred by cancer cell-derived exosomes to vascular endothelial cells (VECs) which enables VEGF to be secreted. Additionally, exosomes can contribute to the promotion of endothelium proliferation by activating the MAPK and Akt signaling pathways, as previously described [74, 94].

### Initiation of metastasis and the pre-metastatic niche

Evidence from recent studies suggests that exosomes have a close association with tumor metastasis, and they could cause the epithelial to mesenchymal transition in lung cancer cells. Messages could be transmitted by the exosomes to initiate the epithelial–mesenchymal transition (EMT) and cause tumor organ metastasis [95]. One of the factors that influences the potential for lung cancer cells to metastasize is the tumor microenvironment [96]. The source and progression of the metastasis could be associated with the exosomes and their contents [97]. Exosomes derived from tumors could be possible mediators of EMT in recipient cells. It was shown by Rahman et al. [98] that exosomes derived from lung cancer cells that metastasized induce expression of vimentin and EMT in receptor human bronchial epithelial cells (HBECs). Additionally, it was shown that exosomes acquired from late-stage lung cancer patient serum had increased vimentin expression, and a more metastatic phenotype was induced in recipient cells. It was suggested that exosomes could trigger the epithelial to mesenchymal transition in lung cancer cells. It has been found that exosomes isolated from different tumor cells that express amphiregulin (AREG) can induce the activation of EGFR in recipient cells, which subsequently affects the microenvironment [99] or promotes bone metastases [100]. Tumor metastasis requires the creation of a pre-metastatic niche [101], where hypoxia and extracellular matrix (ECM) remodeling assist with the formation of this niche [102].

## Exosomes as potential biomarkers

As previously stated, in comparison to standard cells, it is possible for exosome formation to be significantly changed in tumor cells. Furthermore, while all types of cells release exosomes, they can be found in abundance in tumor cells. The upregulation of exosome levels has been detected in the bodily fluids of patients with lung cancer, suggesting that exosomes play a critical role in the development and progression of lung cancer. A growing body of evidence suggests that key exosomal cargo that has been significantly modified under cancerous conditions can act as a biomarker for the diagnosis, prognosis, and prediction of lung cancer [103] (Fig. 1b). Resistance to EGFR-TKIs has also been linked to exosomal cargo. Liu et al. [104] showed that exosomes from cells carrying T790M mutation can induce resistance to gefitinib in sensitive cells via activation of the PI3K/AKT signaling pathway. Exosomal transfer of wild-type EGFR has been described to confer osimertinib resistance by activating this same pathway [105]. Interestingly, exosome cargo can also play a role in reversing gefitinib resistance as shown by Chen et al. [106]. They described exosomal miR-7 as an actor of chemoresistance to gefitinib through the promotion of YAP phosphorylation, an effector of the Hippo pathway. Those studies imply potential novel resistant mechanisms to EGFR-TKIs in NSCLC via exosomes.

Existing studies on exosome-related biomarkers have largely concentrated on miRNAs and proteins derived from exosomes [107–110]. However, more research is now focusing on exosomal EGFR and the role it plays in the pathogenesis and progression of lung cancer. To improve survival rates and to deliver more effective treatment for patients with early-stage cancer, it is essential that the mutation profile of the tumor is monitored as early as possible.

The majority of studies on noninvasive biomarkers of cancer have focused on circulating tumor cells (CTCs), circulating tumor DNA (ctDNA), and circulating free DNA (cfDNA) as well as additional relevant biomarkers, including circulating microRNAs, circulating RNA, platelets, plasma/serum metabolites, or exosomes. A significant amount of cfDNA originates from normal body cells, and only a small proportion (1–3%) of the circulating cfDNA in a patient is associated with tumors, derived from primary tumors, metastatic sites, or CTCs, and is known as ctDNA [111]. Thus far, the detection of EGFR has been primarily based on nucleic acid sourced from ctDNA and is currently being employed in clinical practice [4, 112]. The first liquid biopsy test to receive market approval from the Food and Drug Administration (FDA) is the Cobas EGFR Mutation Test v2® (Roche Diagnostics Inc.) [113, 114]. This test allows the mutations that exist in the cfDNA fraction to be analyzed, such as exon 19 deletions or exon 21 (L858R) substitution mutations in the EGFR gene; however, its ability to detect *EGFR*-T790M is constrained by 58% sensitivity and 80% specificity. Nevertheless, liquid biopsies using cfDNA can be problematic, largely due to the properties of ctDNA and the detection constraints of the methods, even with assay platforms of the highest sensitivity [115–118]. Moreover, there is increasing evidence of heterogeneity between different metastatic sites within the same patient. In addition, concentrations of cfDNA vary enormously between individuals and their physiopathological conditions, and early-stage cancers display a low amount of cfDNA similar to healthy subjects [119], while the fraction of ctDNA fragments in the total cfDNA augments with tumor burden and multiple metastatic sites [120].

To resolve these problems, three recently conducted studies revealed the advantages of combining the mutations identified exosomal nucleic acids (exoNA) with cfDNA for detecting mutations [121–123]. Moreover, the evidence presented by Fernando et al. [124] showed that a significant proportion (93%) of plasma cfDNA is localized in exosomes. Krug et al. [123] showed the utility of exosomal RNA for identifying somatic mutations originating from tumors. Their study involved the parallel screening of exosomal RNA and cfDNA from a cohort composed of 84 NSCLC patients positive for EGFR (stage IIIB, IV). The research findings indicated that exosomal RNA exhibited considerably more sensitivity with regard to the detection of activating *EGFR* mutations (98%) and *EGFR*-T790M (90%) in comparison to corresponding cfDNA (82% and 84% for activating EGFR mutations and *EGFR*-T790M, respectively).

Furthermore, it was shown by Castellanos-Rizaldos et al. [125] that combining exoRNA/DNA and cfDNA for the purpose of detecting T790M offered increased sensitivity and specificity in comparison to historical cohorts in which only cfDNA was used. The researchers published another study in which they designed a qPCR-based test (ExoDx *EGFR*) capable of interrogating a panel of 29 mutations in the *EGFR* gene, which included the activating and resistance mutations utilizing exosomal RNA/DNA and cfDNA obtained from the plasma of a cohort consisting of 110 NSCLC patients According to the assay’s performance results, its general sensitivity was 90% for L858R, 83% for T790M, and 73% for exon 19 deletion with corresponding specificities of 100%, 100%, and 96%, respectively [122]. This could additionally be beneficial for preventing needless tumor biopsies to test for EGFR mutation. Targeting exoNA has increased sensitivity and specificity in the detection of EGFR mutation though the type of detection platform used (Cobas, NGS, ddPCR, ARMS-PCR, etc.) plays a major role in those factors (Table 1).

**Table 1:** Detection of EGFR mutation in exosomal nucleic acids

Techniques	Patients	Mutation	Accuracy		Ref
			Sensitivity (%)	Specificity (%)	
ddPCR	47 NSCLC patients	EGFR activating and T790M mutations	76.5	100	Kim et al. [126]
Cobas®	84 patients from the TIGER-X trial	EGFR activating and T790M mutations	98 for EGFR activating 90 for T790M	94 for L858R 100 for del19	Krug et al. [123]
ddPCR	102 NSCLC patients with biopsy-confirmed T790M positive and 108 patients T790M negative	T790	92	89	Castellanos-Rizaldos et al. [125]
ExoDx *EGFR*	110 NSCLC patients	Panel of 29 mutations in *EGFR* gene	90 for L858R 83 for T790M 73 for del19	100 for L858R 100 for T790M 96 for del19	Castellanos-Rizaldos et al. [122]
ARMS-PCR	284 NSCLC patients	EGFR activating and T790M mutations	27.5	96.6	Wan et al. [127]
NGS	9 patients with EGFR mutation in tissues	T790	75	100	Möhrmann et al. [121]

ddPCR*,* droplet digital PCR; ARMS-PCR*,* amplification-refractory mutation system based PCR assay; NGS*, next-generation sequencing*

As mentioned earlier, miRNAs are highly abundant in exosomes, could regulate the stability or translation of targeted mRNA, and are key factors altering the lung cancer microenvironment, potentially enabling progression, invasion, angiogenesis, metastasis, and drug resistance [56, 110, 128, 129]. Exosome structures prevent miRNAs from being degraded, which is conducive to clinical detection. For lung cancer patients, both circulating exosomes and exosomal miRNA have higher concentrations in comparison to those detected in control patients [35]. Hence, exosomal miRNAs can potentially be employed as a tool for diagnosing, predicting, and prognosing lung cancer and are considered to be possible suitable noninvasive tools for both diagnosing cancer early and as therapeutic targets due to the fact that they include critical information in signaling pathways associated with the biological responses of tumors [130].

Various studies have determined the potential diagnostic ability of tumor biomarkers including miR-139-5p, miR-200b-5p, miR-378a, miR-126, and miR-379 for differentiating between lung cancer and healthy previous smokers. Furthermore, it was determined that six exosomal miRNAs (miR-151a-5p, miR-30a-3p, miR-200b-5p, miR-629, miR-100, and miR-154-3p) were differently expressed in lung adenocarcinoma and granuloma [131–133]. The sensitivity and specificity of these six microRNAs were 96% and 60%, respectively, and they showed high potential for clinical application in terms of differentiating between lung adenocarcinomas and granulomas.

In the current era in which medicine is becoming increasingly personalized, the creation of beneficial and powerful instruments that provide minimal or noninvasive, highly sensitive, simple, quick, and inexpensive diagnosis of cancer is essential. Numerous liquid biopsy biosensors that are intended to detect exosomal proteins, DNA, RNA, and miRNA as biomarkers for the purpose of screening, diagnosing, and prognosticating cancer have been proposed including immunofluorescence biosensor, colorimetric biosensor, surface plasmon resonance (SPR) biosensor, surface-enhanced Raman scattering (SERS) biosensor, electrochemical biosensor, nuclear magnetic resonance (NMR) biosensor, PCR, and next-generation sequencing (NGS) [134]. They could act in parallel or as supplementary tests for screening and diagnosing cancer, personalizing therapy, monitoring response to treatment, and prognosticating.

However, a limitation of some of the studies of exosomes as biomarkers includes the retrospective nature of them, a relatively limited follow-up time, and in some instances a small cohort of patients which may not completely reflect real-life clinic situation. Furthermore, exoNA are often studied in combination with cfDNA [121–123, 125], which may interfere with the interpretation of exosomes alone as biomarkers for diagnosing, predicting, and prognosticating in lung cancer. Additional studies in larger cohorts are needed to prove the robustness of exosome application as biomarkers.

There is increasing interest in the therapeutic potential of exosomes. Notably, a multicenter investigation, the EXTRA study [135] is ongoing, which aims to evaluate exosomes and their contribution to EGFR-TKI resistance mechanisms. This trial will utilize genomic, proteomic, epigenomic, and metabolomic data to determine the different mechanisms underlying treatment efficacy and the development of resistance to afatinib of non-small cell lung cancer patients expressing EGFR.

However, the use of exosomes as biomarkers and/or therapeutic agents still faces major challenges, including the lack of standardization in isolation and purification techniques. Establishing a quick, accurate, and low-cost common method is crucial for clinical application. Moreover, the heterogeneity of the exosome population is secreted in body fluids as they originate from a variety of different cell types, making it challenging to determine their tissues of origin. Therefore, a precise understanding of exosome characteristics and profiles is essential.

## Conclusion

Exosomes may be used to identify somatic mutations of tumors from liquid biopsies [8, 69], with *EGFR* and its signaling network proteins frequently detected in exosomes from NSCLC patients, making them potential biomarkers for cancer research and clinical use [75, 76, 78]. New platforms display promising perspectives in their ability to detect EGFR mutations in liquid biopsy. One of the most noteworthy is the recently and first approved FDA cfDNA-based plasma test for EGFR mutations [113]. Promisingly, the measurement of exoNA may expand the utility of exosomes as potential diagnostic and prognostic tools in EGFR-mutated cancers as they could provide a more complete assessment of the evolving tumor and response to targeted therapies.
